# Right ventricular outflow tract isolation for biventricular tachycardia following myocardial infarction

**DOI:** 10.1016/j.hrcr.2024.08.025

**Published:** 2024-08-31

**Authors:** Rintaro Hojo, Takafumi Sasaki, Koichiro Yamaoka, Hirofumi Kujiraoka, Tomoyuki Arai, Masao Takahashi, Seiji Fukamizu

**Affiliations:** 1 Department of Cardiology, Tokyo Metropolitan Hiroo Hospital, Tokyo, Japan

**Keywords:** Biventricular tachycardia, Right ventricular outflow tract isolation, Catheter ablation, Bipolar ablation, Myocardial infarction

**Key Teaching Points**

• For a circuit of biventricular tachycardia to be established, there must be an extensive scar area in the ventricular septum. The scar area is formed by sarcoidosis, myocardial infarction, and prior catheter ablation.
• In biventricular tachycardia, disconnection of conduction between the ventricles is a therapeutic option. Isolation of the right ventricular outflow tract may be effective when it is difficult to disconnect the interventricular conduction due to multiple connection sites.
• If interventricular conduction cannot be blocked by ablation from endocardial side, bipolar ablation between the endocardium and the epicardium might be effective.

## Introduction

Ventricular tachycardia (VT) involving circuits of both ventricles have been reported as cases of biventricular VT (biVT).^1^ Owing to its rarity, however, the optimal ablation site for biVT remains unclear. In this report, we present a case of biVT after acute myocardial infarction. Ablation was done to isolate the right ventricular outflow tract (RVOT), successfully interrupting the biVT circuit.

## Case report

A 52-year-old man presented to our hospital with chest pain. On arrival, he experienced cardiac arrest and underwent successful resuscitation with percutaneous cardiopulmonary support. Coronary angiography revealed occlusion of the left anterior descending artery necessitating revascularization with stenting. On day 5, the patient developed ventricular fibrillation and VT after withdrawal of percutaneous cardiopulmonary support, which were temporarily resolved with amiodarone infusion. Catheter ablation (CA) was performed on day 14 for VT storm. Electroanatomic mapping demonstrated a low-voltage area in the anterior and septal regions of the left ventricle (LV). During VT, diastolic potentials were recorded in the septal LV, obtaining a good pace map for the VT. Nonsustained nature of the VT made it difficult to determine its electrophysiological characteristics. Noninducibility of VT was confirmed following CA of the septal area.

**Figure 1:**
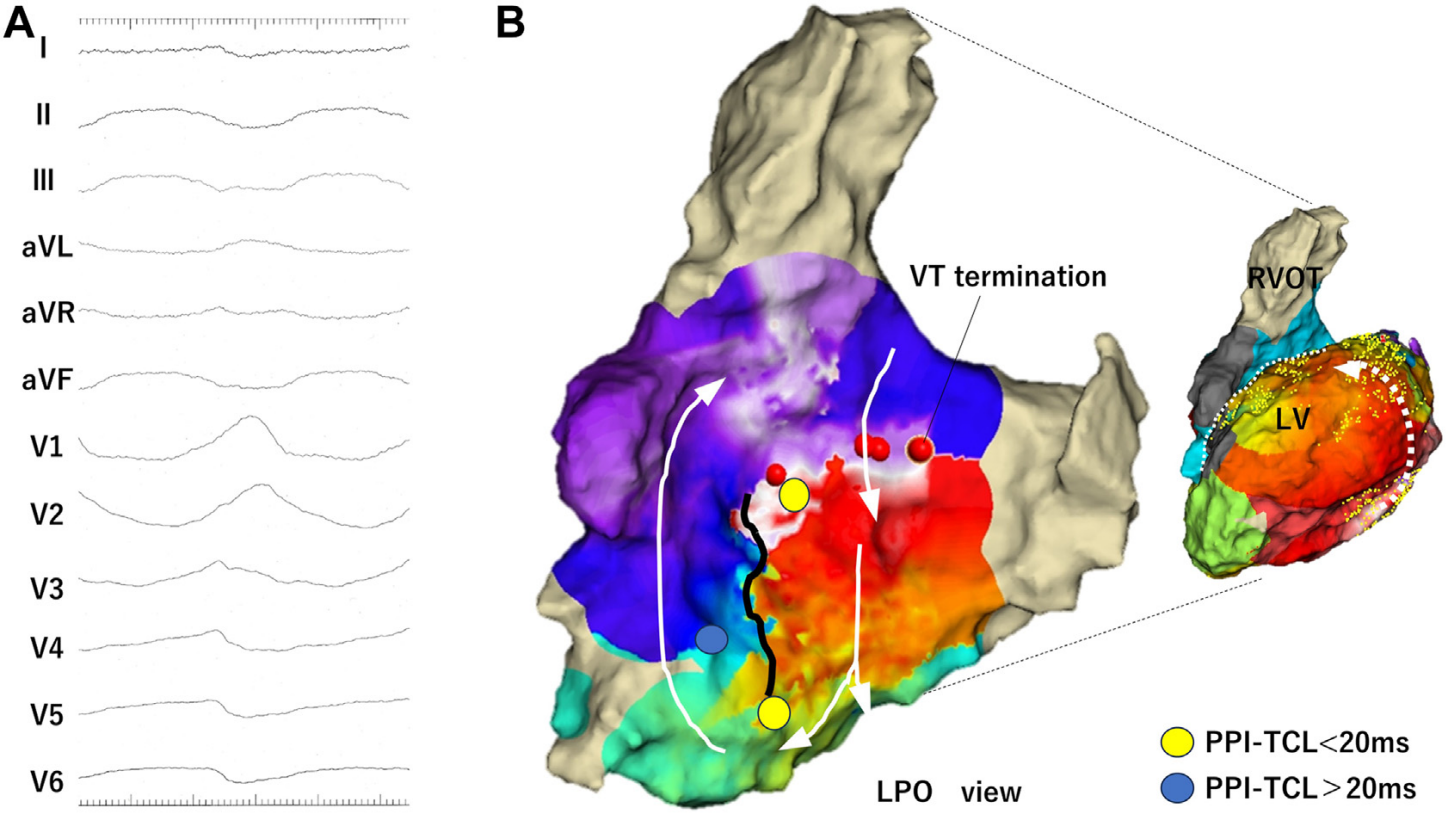
**A:** Sustained ventricular tachycardia (VT1) demonstrating an right bundle branch block morphology with a tachycardia cycle length (TCL) of 285 milliseconds. **B:** The activation map during VT1 in the left ventricle (LV) shows a focal pattern that does not cover the TCL. The activation map of the right ventricular septum shows clockwise propagation around the line of the block. Post-pacing interval (PPI) shows the corridor between the line of the block and the tricuspid valve, including the VT1 circuit. Linear ablation from the line of the block to the tricuspid valve successfully terminated VT1. LPO = left posterior oblique view; RVOT = right ventricular outflow tract.

On day 15, CA was performed for the recurrence of sustained VT (tachycardia cycle length [TCL], 290 milliseconds) with right bundle branch block (RBBB) and superior axis morphology, termed as *VT1* (Figure 1A). Activation mapping using Ensite X (Abbott, Plymouth, MN) revealed a focal activation pattern originating from the LV posterior wall that did not cover the TCL. Meanwhile, the activation map and post-pacing interval (PPI) of the right ventricular septum suggested the involvement of the basal region in the tachycardic circuit (Figure 1B and Supplemental Video 1A). Linear ablation along the line of the block towards the tricuspid valve successfully terminated VT1.

**Figure 2:**
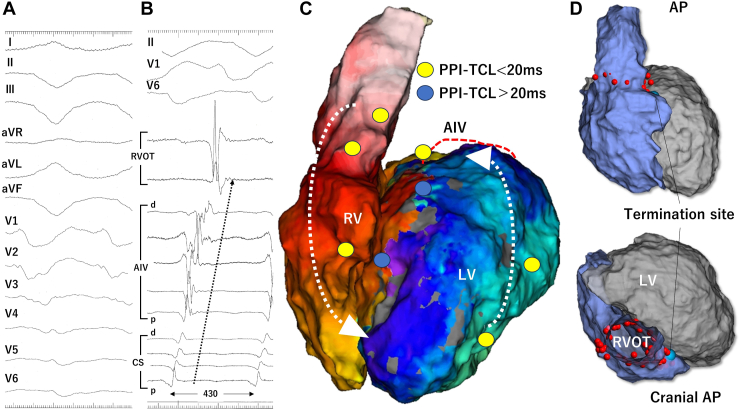
An electrocardiogram and activation map of the recurred ventricular tachycardia (VT2) with a tachycardia cycle length (TCL) of 430 milliseconds. **A:** Twelve-lead electrocardiogram during VT2 shows an right bundle branch block morphology. **B:** Intracardiac electrocardiogram during VT2. In addition to both the right ventricle (RV) and left ventricle (LV), insertion of an electrode catheter in the anterior interventricular vein (AIV) enabled the recording of the total TCL. **C:** Activation map during VT2 demonstrating counterclockwise propagation in the left anterior oblique view. Post-pacing intervals (PPIs) in the yellow circle are similar to those in the TCL. The PPIs in the *blue circles* are longer by more than 20 milliseconds compared with those in the TCL. **D:** The right ventricular outflow tract (RVOT) isolation line and VT termination site are shown in anterior posterior view. AP = anterior posterior view; CS = coronary sinus.

Extrastimulation from the right ventricle (RV) induced VT (TCL, 430 milliseconds) with left bundle branch block and superior axis morphology (termed *VT2*) (Figure 2A). Activation maps of the RV and LV demonstrated cranial-to-caudal propagation in the RV and posterior-to-anterior activation in the LV (Figure 2C and Supplemental Video 1B). The PPI–TCL differences were 0 milliseconds at the RVOT, 10 milliseconds at the anterior RV, 20 milliseconds at the inferior LV, and 10 milliseconds at the posterior LV. These findings suggested biventricular involvement in the VT circuit. In addition, the placement of an electrode catheter in the anterior interventricular vein (AIV) allowed the documentation of the total TCL (Figure 2B). However, ablation attempts at the earliest site of the RVOT, lateral LV, or endocardium at the LV summit failed to terminate VT2.

**Supplemental Figure 1:**
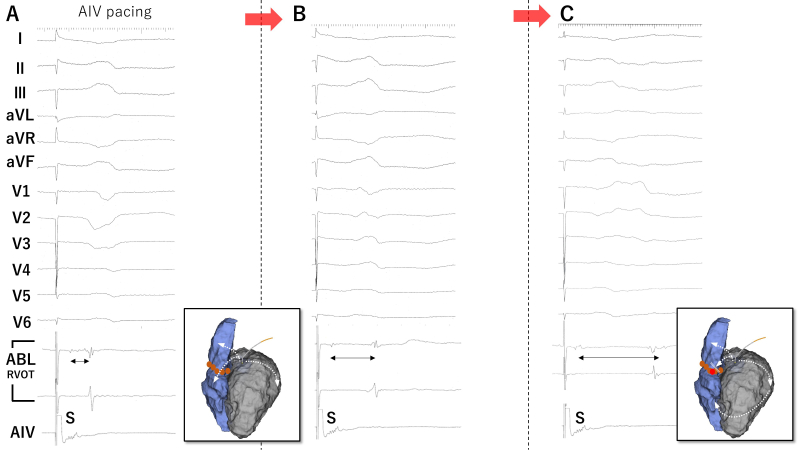
After ventricular tachycardia 2 (VT2) termination, conduction between the right ventricle and the left ventricle was assessed with pacing from the anterior interventricular vein (AIV). Change in 12-lead electrogram and intracardiac electrocardiogram during the AIV pacing are shown. A: Conduction from the AIV was observed on the cranial and caudal sides of the isolation line, resulting in double potential on the isolation line. B: When ablation (ABL) was added to block the conduction below the isolation line, the interval of double potential gradually prolonged and QRS morphology changed to right bundle branch block. C: Finally, the QRS width was also prolonged, and the conduction from the AIV to the caudal side of the isolation line was blocked. RVOT = right ventricular outflow tract.

Because the above treatments were ineffective, we opted for circumferential linear ablation to isolate the RVOT, which successfully terminated the biVT (Figure 2D). Following VT2 termination, additional ablation was performed during AIV pacing. Conduction from the AIV was observed on both cranial and caudal sides of the encircling line, resulting in a fused double potential on the encircling line (Supplemental Figure 1A). Further ablation below the isolation line progressively increased the double potential interval, increasing the width of the QRS morphology to form an RBBB (Supplemental Figure 1B and 1C). These changes indicated the successful conduction block creation to the RV during pacing from the AIV.

**Figure 3:**
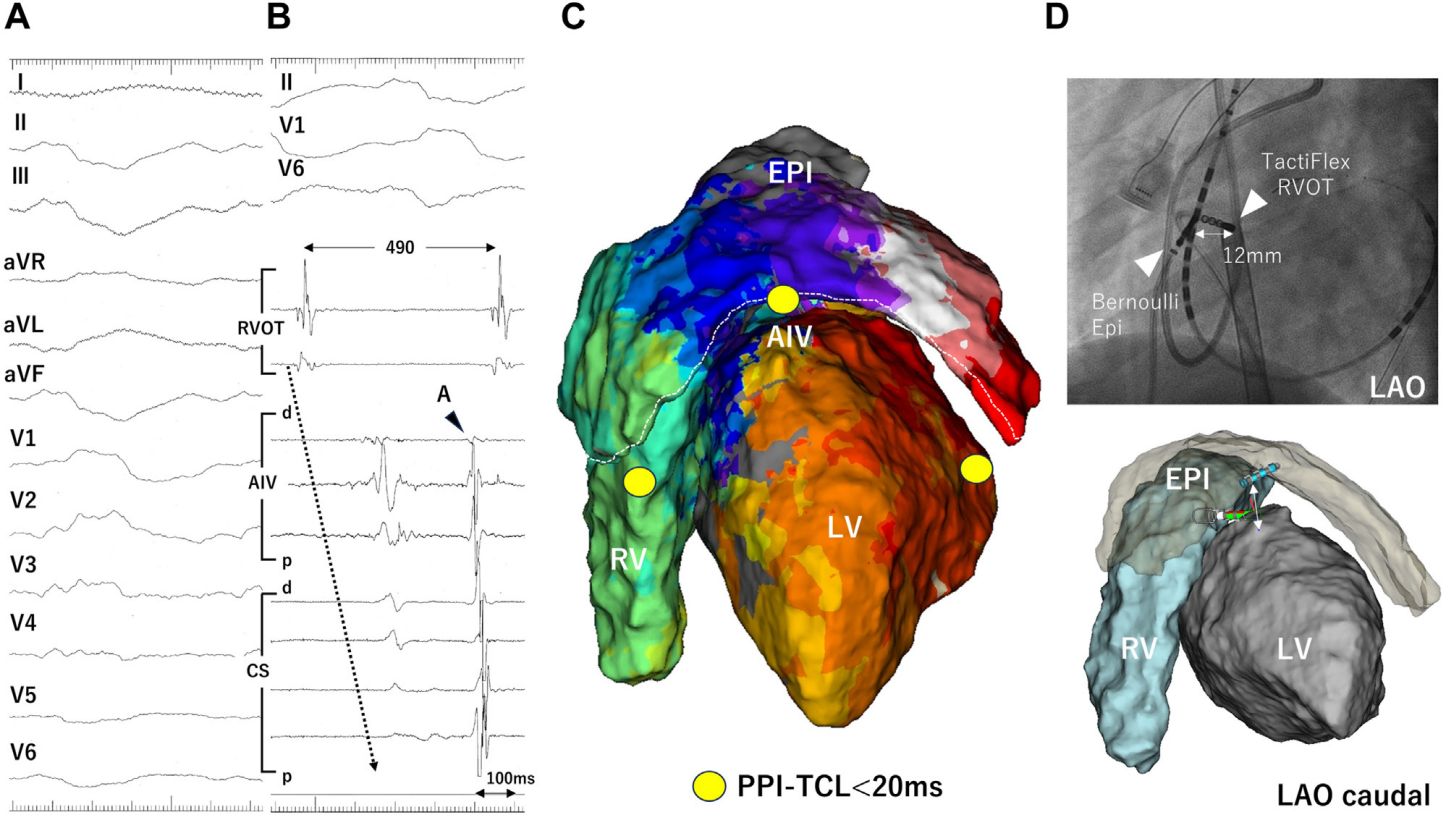
An electrocardiogram and activation map during the recurred ventricular tachycardia (VT3) with a TCL of 490 milliseconds. **A:** Twelve-lead electrocardiogram during VT3 shows an right bundle branch block morphology. **B:** An intracardiac electrocardiogram of VT3. **C:** Activation map during VT3 demonstrating clockwise propagation in the left anterior oblique (LAO) view, which is the reverse activation of VT2. Post-pacing intervals (PPIs) in the *yellow circle* are similar to those in the tachycardia cycle length (TCL). **D:** The catheter position during bipolar ablation of VT3. The active catheter, TactiFlex (Abbott, Plymouth, MN), is located in the right ventricular outflow tract (RVOT), and the ground catheter, Bernoulli (Japan Lifeline, Tokyo, Japan) is inserted into the epicardium (EPI). AIV = anterior interventricular vein; CS = coronary sinus.

Approximately 1 week later, the patient developed recurrence of VT (TCL, 490 milliseconds) with RBBB morphology (Figure 3A) (termed *VT3*). In the re-do procedure including epicardial mapping, activation maps of the endocardial and epicardial regions of the RV and LV revealed a reverse activation pattern of VT2 (Figure 3C). The PPIs closely resembled the TCL in the anterior RVOT, the AIV, and the lateral LV.

**Supplemental Figure 2:**
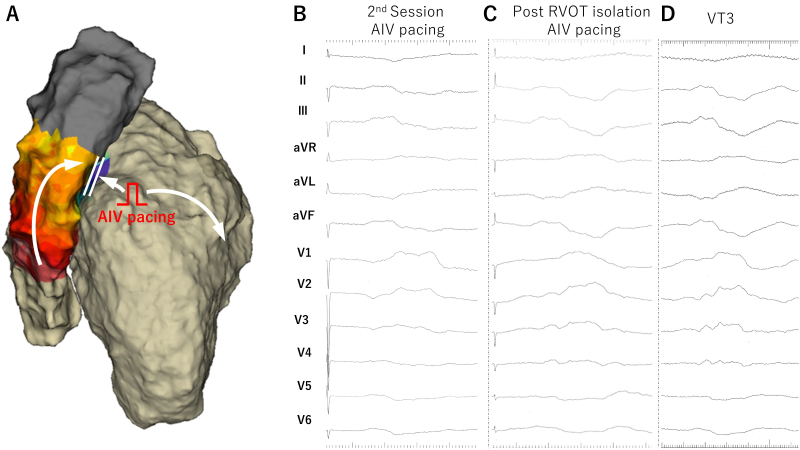
After ventricular tachycardia 3 (VT3) termination, conduction between the right ventricle and the left ventricle was assessed with pacing from the anterior inter-ventricle vein. A: Activation map of right ventricle during the anterior interventricle vein (AIV) pacing demonstrated the caudal to cranial propagation. B: The QRS morphology during AIV pacing in the second session. C, D: The QRS morphology after VT3 termination changed wider than the QRS morphology in the second session, and was similar to the QRS morphology of VT3. RVOT = right ventricular outflow tract.

Although reconduction between the RVOT and LV was suspected to underlie VT3, further ablation from the endocardial side of the RVOT and bipolar ablation between the RVOT and the LV failed to achieve VT termination. Left coronary angiography was performed subsequently, and bipolar ablation between the RVOT and epicardium successfully terminated VT3 (Figure 3D). Following CA for VT3, the RVOT activation map during AIV pacing demonstrated caudal-to-cranial propagation (Supplemental Figure 2A). The post-ablation QRS width during the AIV pacing (Supplemental Figure 2C) was wider compared with that observed in the second session (Supplemental Figure 2B), similarly resembling the QRS morphology of VT3 (Supplemental Figure 2D). In addition, any potential was not observed in the RVOT, confirming complete RVOT isolation. Finally, programmed stimulation confirmed noninducibility of any VTs.

## Discussion

To the best of our knowledge, only 1 case report on biVT has been published, which describes it as a case of cardiac sarcoidosis.^1^ Our case presents biVT occurring after acute myocardial infarction, demonstrating propagation in both direction, which was revealed by epicardial mapping. Extensive scarring tissue in the ventricular septum is considered essential for the establishment of a biVT circuit. In this case, the infarcted area developed remodeling and VT on day 14 of admission. In addition, the septal lesion that developed during the first session likely overlapped the infarcted area and created a larger circuit of VT. For the second session, a conduction block was created from the right ventricular septum to the tricuspid valve. As such, a larger circuit was established, which included the RVOT and prolonged the TCL, thereby resulting in biVT.

Disruption of the interventricular conduction is a potential therapeutic strategy for biVT. A previous report described the successful ablation of the RVOT endocardium, achieving noninducibility of VT.^1^ If the scar area extends to the RVOT, as reported previously, a few points of ablation may be sufficient to block the tachycardia circuit. However, if the ROVT is less compromised, as in the present case, there is broad conduction from the epicardial side to the RVOT, which may make it difficult to interrupt the circuit. In such cases, RVOT isolation may be effective. RVOT isolation has been reported to be effective for VT originated from the RVOT that was not inducible during catheterization.^2^ When performing RVOT isolation, the isolation area should be determined to include multiple interventricular conductions. After RVOT isolation, it is necessary to check for residual conduction by pacing from the AIV.

The appearance of an RBBB waveform during the AIV pacing suggests slower conduction to the RV from the AIV (Supplemental Figure 1). If conduction to the RV from the AIV is completely disrupted, the waveform would be similar to that of VT3 (Supplemental Figure 2). If conduction from the epicardium to the RVOT is within the site of RVOT isolation, similar changes would occur, making it impossible to distinguish between RVOT isolation and AIV–RVOT conduction block. In both situations, a conduction block occurs in the circuit of biVT. In this case, the RVOT isolation was finally confirmed by voltage map. If interventricular conduction cannot be blocked by ablation from the endocardial side, bipolar ablation between the endocardium and the epicardium might be effective, as demonstrated in this case.

## Conclusion

Anteroseptal myocardial infarction complicated with ablation-induced ventricular septal scar can establish a biVT circuit with propagation in both directions. Isolation of the RVOT, the site of interventricular conduction attachment, may be an effective treatment for such cases.

## Disclosures

The authors have no conflicts of interest to disclose.

## Appendix Supplementary Data

Supplemental Video 1 **A:** Propagation map of ventricular tachycardia (VT) 1 in the right ventricular septum. **B:** Propagation map of the right and left ventricle during VT 2 showed clockwise activation in left anterior oblique view. C: Propagation map of right and left ventricle and epicardium during VT 3 demonstrated reverse activation of VT2.

## Footnotes

Appendix Supplementary data associated with this article can be found in the online version at https://doi.org/10.1016/j.hrcr.2024.08.025.
